# Gene Dosage- and Age-Dependent Differential Transcriptomic Changes in the Prefrontal Cortex of *Shank2*-Mutant Mice

**DOI:** 10.3389/fnmol.2021.683196

**Published:** 2021-06-11

**Authors:** Seungjoon Lee, Hyojin Kang, Hwajin Jung, Eunjoon Kim, Eunee Lee

**Affiliations:** ^1^Department of Biological Sciences, Korea Advanced Institute for Science and Technology (KAIST), Daejeon, South Korea; ^2^Division of National Supercomputing, KISTI, Daejeon, South Korea; ^3^Center for Synaptic Brain Dysfunctions, Institute for Basic Science (IBS), Daejeon, South Korea; ^4^Department of Anatomy, School of Medicine, Yonsei University, Seoul, South Korea

**Keywords:** autism spectrum disorder, Shank2, prefrontal cortex, transcript, RNA-seq, dosage-dependence, age-dependence

## Abstract

Shank2 is an abundant postsynaptic scaffolding protein that is known to regulate excitatory synapse assembly and synaptic transmission and has been implicated in various neurodevelopmental disorders, including autism spectrum disorders (ASD). Previous studies on *Shank2*-mutant mice provided mechanistic insights into their autistic-like phenotypes, but it remains unclear how transcriptomic patterns are changed in brain regions of the mutant mice in age- and gene dosage-dependent manners. To this end, we performed RNA-Seq analyses of the transcripts from the prefrontal cortex (PFC) of heterozygous and homozygous *Shank2*-mutant mice lacking exons 6 and 7 at juvenile (week 3) and adult (week 12) stages. Juvenile heterozygous *Shank2*-mutant mice showed upregulation of glutamate synapse-related genes, downregulation of ribosomal and mitochondrial genes, and transcriptomic changes that are opposite to those observed in ASD (anti-ASD) such as upregulation of ASD_down (downregulated in ASD), GABA neuron-related, and oligodendrocyte-related genes. Juvenile homozygous *Shank2* mice showed upregulation of chromatin-related genes and transcriptomic changes that are in line with those occurring in ASD (pro-ASD) such as downregulation of ASD_down, GABA neuron-related, and oligodendrocyte-related genes. Adult heterozygous and homozygous *Shank2*-mutant mice both exhibited downregulation of ribosomal and mitochondrial genes and pro-ASD transcriptomic changes. Therefore, the gene dosage- and age-dependent effects of *Shank2* deletions in mice include differential transcriptomic changes across distinct functional contexts, including synapses, chromatin, ribosomes, mitochondria, GABA neurons, and oligodendrocytes.

## Introduction

Shank2 (also known as ProSAP1) is an abundant scaffolding protein known to regulate excitatory synapse assembly and synaptic transmission, plasticity, and signaling (Du et al., 1998; Boeckers et al., 1999; Lim et al., 1999; Naisbitt et al., 1999); reviewed in Boeckers et al. (2002); Grabrucker et al. (2011); Sala et al. (2015); Mossa et al. (2017); and Sheng and Kim (2000, 2011). Shank2 has also been implicated in various neurodevelopmental and neuropsychiatric disorders, including autism spectrum disorders (ASD), intellectual disability, developmental delay, and schizophrenia (Berkel et al., 2010, 2011; Pinto et al., 2010; Wischmeijer et al., 2011; Leblond et al., 2012, 2014; Prasad et al., 2012; Rauch et al., 2012; Sanders et al., 2012; Chilian et al., 2013; Liu et al., 2013; Schluth-Bolard et al., 2013; Guilmatre et al., 2014; Costas, 2015; Peykov et al., 2015a,b; Homann et al., 2016; Mossa et al., 2017; Yuen et al., 2017; Bai et al., 2018; Lu et al., 2018; Aspromonte et al., 2019; Zhou et al., 2019; Satterstrom et al., 2020; Wang et al., 2020). Mice carrying *Shank2* mutations have enabled researchers to gain important mechanistic insights into how *Shank2* deletion leads to various disease-related deficits at the molecular, synaptic, circuit, and behavioral levels (Schmeisser et al., 2012; Won et al., 2012; Ey et al., 2013, 2018; Ferhat et al., 2016; Ha et al., 2016; Ko et al., 2016; Peter et al., 2016; Lim et al., 2017; Pappas et al., 2017; Sato et al., 2017, 2020; Yoon et al., 2017; Heise et al., 2018; Kim et al., 2018; Lee S. et al., 2018; Wegener et al., 2018; Chung et al., 2019; de Chaumont et al., 2019; Han et al., 2020; Lee et al., 2020; Grabrucker et al., 2021; Horner et al., 2021); reviewed in Boeckers et al. (2002); Grabrucker et al. (2011); Jiang and Ehlers (2013); Guilmatre et al. (2014); Sala et al. (2015); Schmeisser (2015); Monteiro and Feng (2017); Mossa et al. (2017); Eltokhi et al. (2018); and Yoo et al. (2014). Many of the mechanistic studies have focused on how synaptic changes lead to behavioral abnormalities, partly because of the known functions of Shank2 at excitatory synapses. However, *Shank2*-mutant phenotypes likely involve various molecular and cellular changes, which should be explored comprehensively in various brain regions and cell types across different developmental stages and *Shank2* gene dosages.

To begin such work, we herein analyzed the transcriptomic patterns in prefrontal cortex (PFC) regions of heterozygous (HT; *Shank2*^+/–^) and homozygous (HM; *Shank2*^–/–^) *Shank2*-mutant mice at juvenile (P21 or 3 weeks) and adult (P84 or 12 weeks) stages (W3-HT, W3-HM, W12-HT, and W12-HM). We chose to analyze the PFC due to its reported implications in cognitive functions, social functions, and ASD (Wang et al., 2011; Yizhar et al., 2011; Lee et al., 2016; Selimbeyoglu et al., 2017; Cao et al., 2018; Lazaro et al., 2019; Levy et al., 2019). We identified differential transcriptomic changes involving multiple biological functions and ASD-related genes, and found that they were distinct between W3-HT and W3-HM mice, but largely similar between W12-HT and W12-HM mice. These analyses also identified the potential involvement of genes associated with various functional contexts, including synapses, chromatin, ribosomes, mitochondria, GABA neurons, and oligodendrocytes.

## Materials and Methods

### Animals

*Shank2* mice lacking exons 6 and 7 have been described (Won et al., 2012) (B6N.129S4-*Shank2*^*tm1Mgle*^/CsbdJ; Jackson 033667). Animals were housed under a 12-h (13:00–01:00) dark/light cycle and were fed *ad libitum*. The animal study was reviewed and approved by the Committee of Animal Research at KAIST (KA2020-99).

### Tissue Preparation for RNA-Seq Analysis

Brains isolated from *Shank2*^±^ and *Shank2*^–/–^ mice (*n* = 5 brains from five mice for W3-HT, five for W3-HM, four for W12-HT, and four for W12-HM) were chilled in ice-cold phosphate-buffered saline solution. Mouse brains were not pooled, and samples from individual mouse brains were handled independently. Coronal brain sections (1 mm thickness) were used to dissect out PFC regions (AP + 2.8 to + 1.1 mm; ML −1 to +1 mm). The dissected tissues were kept in RNAlater solution (Ambion) at room temperature for 30 min and then kept at –70°C. Total RNA concentration was calculated by Quant-IT RiboGreen (Invitrogen, R11490). To determine the integrity of total RNAs, samples were run on the TapeStation RNA screentape (Agilent). Only high-quality RNA preparations, with RIN greater than 7.0, were used for RNA library construction. A library was prepared with 1 μg of total RNA for each sample by Illumina TruSeq mRNA Sample Prep kit (Illumina). The first step in the workflow involved purifying the poly-A-containing mRNA molecules using poly-T oligo-attached magnetic beads. Following purification, the mRNA is fragmented into small pieces using divalent cations under elevated temperature. The cleaved RNA fragments are copied into first-strand cDNAs using SuperScript II reverse transcriptase (Invitrogen) and random primers, which was followed by second-strand cDNA synthesis using DNA Polymerase I and RNase H. These cDNA fragments then went through an end repair process, the addition of a single “A” base, and then ligation of the indexing adapters. The products were then purified and enriched with PCR to create the final cDNA library. The libraries were quantified using qPCR according to the qPCR Quantification Protocol Guide (KAPA Library Quantification kits for Illumina Sequencing platforms) and qualified using the TapeStation D1000 ScreenTape (Agilent Technologies). Indexed libraries were then submitted to an Illumina NovaSeq (Illumina), and the paired-end (2 × 100 bp) sequencing was performed by Macrogen.

### RNA-Seq Analysis

Transcript abundance was estimated with Salmon (v1.1.0) (Patro et al., 2017) in Quasi-mapping-based mode onto the Mus musculus genome (GRCm38) with GC bias correction (–gcBias). Quantified gene-level abundance data was imported to R (v.3.5.3) with the tximport (Soneson et al., 2015) package, and differential gene expression analysis was carried out using R/Bioconductor DEseq2 (v1.22.2) (Love et al., 2014). Some genes were filtered out when their adjusted *p* values were null value, failing to pass the independent filtering step in DESeq2, and when an HGNC (HUGO Gene Nomenclature Committee) human gene did not exist for a given mouse gene. For independent quality control, the percentage of mapped reads (y-axis) was plotted against the number of mapped reads (x-axis). The scatter plots were drawn using MultiQC tools and the statistics data from Salmon. Principle component analysis (PCA) was performed for the regularized log transform (rlog) of the normalized counts using plotPCA tools implemented in DEseq2. Normalized read counts were computed by dividing the raw read counts by size factors and fitted to a negative binomial distribution. The *P* values were adjusted for multiple testing for each developmental stage and comparisons with the Benjamini–Hochberg correction. Genes with an adjusted *P* value of less than 0.05 were considered as differentially expressed. Volcano plots were generated using the R ggplot2 (v.3.1.1) package. The Gene Ontology (GO) enrichment analyses were performed using DAVID software (version 6.8) (Huang da et al., 2009) on the human background. Mouse gene names were converted to human homologs using the Mouse Genome Informatics (MGI) database^1^.

### Gene Set Enrichment Analysis

Gene Set Enrichment Analysis (GSEA)^2^ (Subramanian et al., 2005) was used to determine whether *a priori*-defined gene sets would show statistically significant differences in expression between WT and *Shank2*-mutant mice. Enrichment analysis was performed using GSEAPreranked (gsea-3.0.jar) module on gene set collections downloaded from Molecular Signature Database (MSigDB) v7.0^3^. GSEAPreranked was applied using the list of all genes expressed, ranked by the fold change and multiplied by the inverse of the *P* value with recommended default settings (1,000 permutations and a classic scoring scheme). The False Discovery Rate (FDR), which adjusts different gene set sizes and multiple testings, was estimated to control the false-positive finding of a given Normalized Enrichment Score (NES) by comparing the tails of the observed and null distributions derived from 1,000 gene set permutations. The gene sets with an FDR of less than 0.05 were considered significantly enriched.

GSEA for biological functions were performed using the entire list of genes with transcriptional changes ranked by *p* values from the four transcript groups (W3-HT, W3-HM, W12-HT, and W12-HM) as inputs (Supplementary Table 1) and the Gene Ontology gene sets in the C5 subdomains at the GSEA site (^2^cellular component, biological process, molecular function; 10,185 gene sets as of now). For ASD-related/risk GSEA, the same inputs and the following gene sets were used (specific gene names in these gene sets are listed in Supplementary Table 9); DEG_Up_Voineagu, Co-Exp_Up_M16_Voineagu, DEG_Down_Voineagu, and Co-Exp_Down_M12_Voineagu (Voineagu et al., 2011; Werling et al., 2016), SFARI genes (all) (Abrahams et al., 2013), SFARI (high confidence, belonging to category 1) (Abrahams et al., 2013)^4^, FMRP targets (Darnell et al., 2011; Werling et al., 2016), DeNovoMis (protein-disrupting or missense rare *de novo* variants) (Iossifov et al., 2014; Werling et al., 2016), DeNovoVariants (protein-disrupting rare *de novo* variants) (Iossifov et al., 2014; Werling et al., 2016), and AutismKB (Autism KnowledgeBase) (Xu et al., 2012; Yang et al., 2018), and cell-type-specific gene sets (Kang et al., 2011).

## Results

### Differentially Expressed Gene Analyses of Heterozygous and Homozygous *Shank2*-Mutant Transcriptomes at Weeks 3 and 12

To determine transcriptomic changes in the PFC of heterozygous (HT; *Shank2*^±^) and homozygous (HM; *Shank2*^–/–^) *Shank2*-mutant mice at juvenile (3 weeks) and adult (12 weeks) stages (W3-HT, W3-HM, W12-HT, and W12-HM mice), relative to wild-type mice (W3-WT and W12-WT; five mice for W3-WT/HT/HM mice and four mice for W12-WT/HT/HM mice), we performed RNA-Seq and analyzed the obtained data. W3 and W12 transcriptomes showed an age-dependent increase in clustering, as shown by PCA (principal component analysis) plots (Supplementary Figure 1).

**FIGURE 1:**
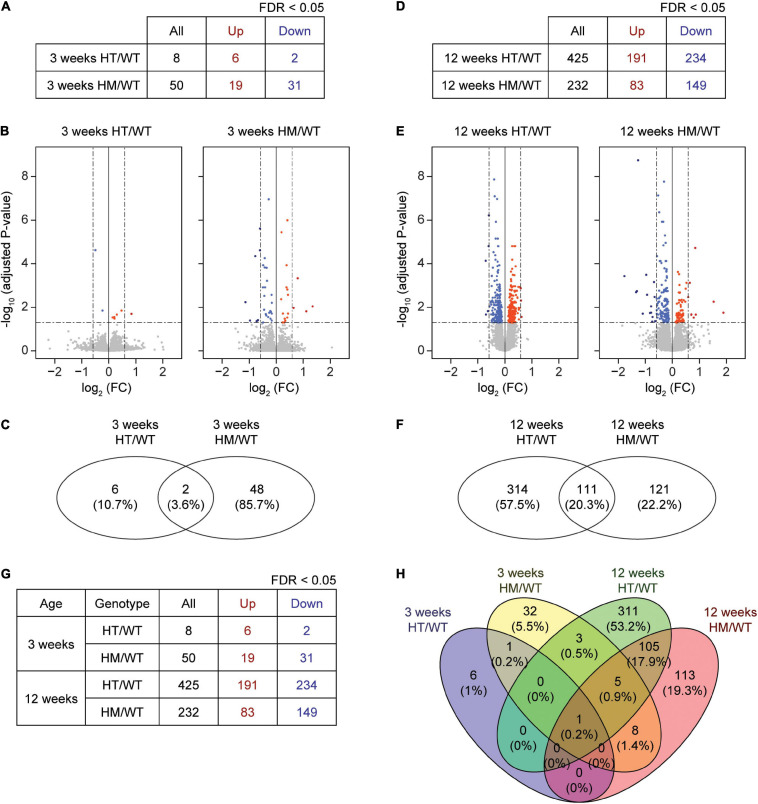
DEG analyses based on RNA-Seq results from W3-HT, W3-HM, W12-HT, and W12-HM *Shank2*-mutant mice. **(A–C)** Summary table, volcano plot, and Venn diagram for DEGs from the prefrontal cortex of W3-HT and W3-HM *Shank2*-mutant mice, relative to WT mice. Red, upregulation; blue, downregulation (*n* = 5 mice for W3-WT/HT/HM; dotted lines indicate adjusted *p* value < 0.05, |FC| > 1.5, of which only adjusted *p* values but not FCs were used to define DEGs). **(D–F)** Summary table, volcano plot, and Venn diagram for the DEGs from the prefrontal cortex of W12-HT and W12-HM *Shank2*-mutant mice, relative to WT mice. Red, upregulation; blue, downregulation (*n* = 4 mice for W12-WT/HT/HM; dotted lines indicate adjusted *p*-value < 0.05, |FC| > 1.5). **(G,H)** Table summarizing all DEGs and a Venn diagram showing the overlaps in DEGs from W3-HT, W3-HM, W12-HT, and W12-HM *Shank2*-mutant mice. Red, upregulation; blue, downregulation.

At week 3, eight and 50 DEGs were identified in the PFC of W3-HT and W3-HM mice, respectively, compared to WT mice at 3 weeks (Figures 1A–C and Supplementary Tables 1, 2; see W3-HT/HM tabs). Only two of the identified DEGs (Shank2 and NFIB; 3.6%) overlapped between 3W-HT and 3W-HM. At week 12, in contrast, we identified many more DEGs in 12W-HT (425; 191 upregulated and 234 downregulated) and 12W-HM (232; 83 upregulated and 149 downregulated) mice compared with W12-WT mice (Figures 1D–F and Supplementary Tables 1, 2; see W12-HT/HM tabs). Of them, 111 DEGs (20.3%) overlapped between 12W-HT and 12W-HM (see W12-HT-HM overlap tab).

A numerical comparison of the four DEG sets (Figure 1G) highlighted that there were age-dependent increases in the total numbers of DEGs in both heterozygous and homozygous *Shank2* mice, with a greater increase seen in heterozygous mice. A Venn diagram plotting the DEG numbers showed that there were small overlaps between W3 and W12 DEGs within the HT and HM groups [0.2% (HT) and 5.2% (HM), respectively], compared to the greater overlaps of HT and HM DEGs at W3 (3.6%) and W12 (20.3%) (Figure 1H).

**FIGURE 2:**
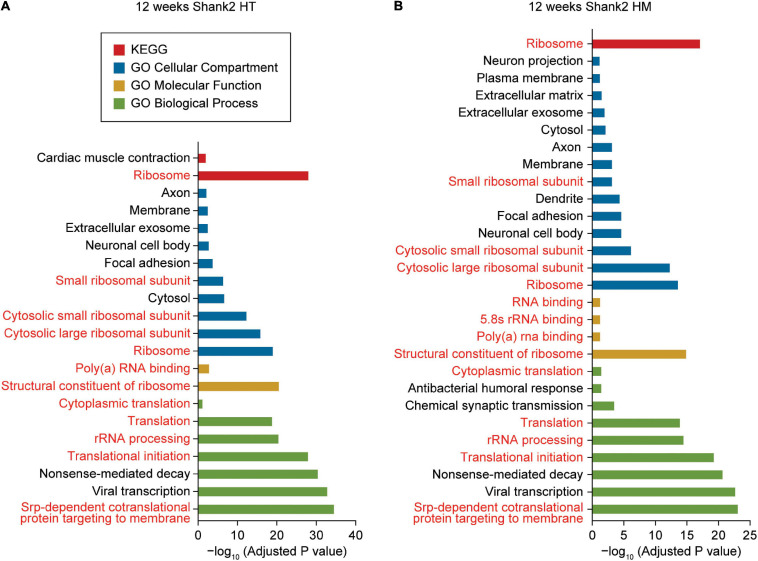
DAVID gene ontology analyses of DEGs from W12-HT and W12-HM *Shank2*-mutant mice. **(A,B)** DAVID gene ontology (GO) analysis of DEGs from W12-HT and W12-HM *Shank2*-mutant mice (adjusted *p*-value < 0.05). Ribosome/translation-related GO terms are indicated in red.

DAVID analysis of W12-HT transcripts (up- and downregulated together) revealed ribosome- and translation-related GO terms in the cellular component (CC), biological process (BP), molecular function (MF), and KEGG (Kyoto Encyclopedia of Genes and Genomes) domains (Figure 2A and Supplementary Table 3). GO terms related to the neuronal cell body, axon, and exosome were overrepresented in the CC domain but not in BP or MF domain.

DAVID analysis of W12-HM transcripts (up and downregulated together) highlighted similar GO terms (ribosome/translation-related and neuronal cell body/axon/ exosome-related) (Figure 2B). The up- and downregulated terms were pooled for these analyses to increase the strength of our analytic results.

### GSEA of Heterozygous and Homozygous *Shank2*-Mutant Transcriptomes at 3 and 12 Weeks for Biological Functions

We additionally performed GSEA, which uses the entire list of genes with transcriptional changes (ranked by *p* values) rather than the relatively small number of DEGs above a particular *p* value or fold change, in order to identify altered biological pathways contributed by modest but coordinate changes in a more reproducible manner (Mootha et al., 2003; Subramanian et al., 2005) (see text footnote 4).

**FIGURE 3:**
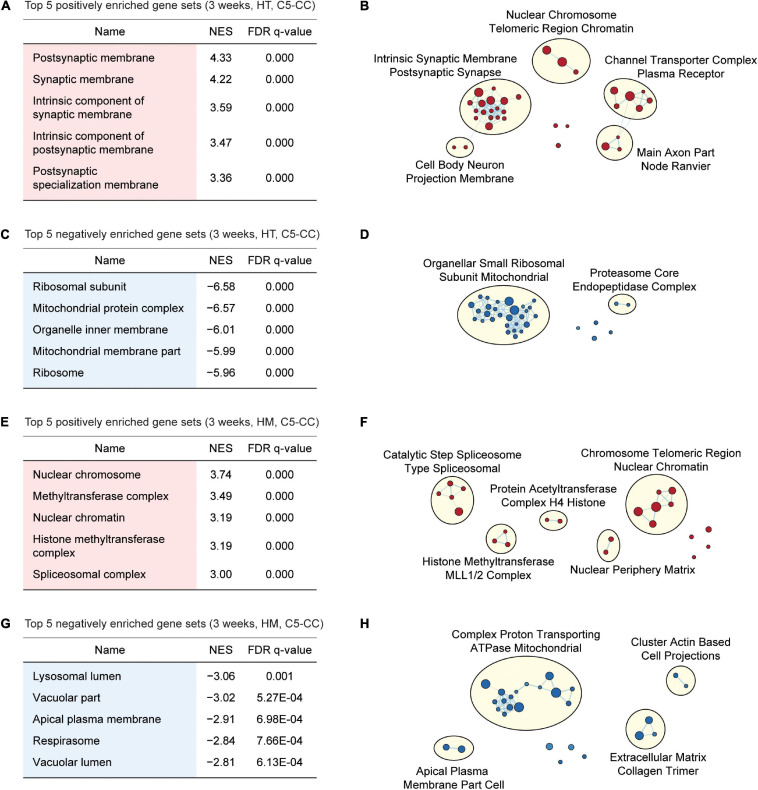
GSEA of transcriptomes from W3-HT and W3-HM *Shank2*-mutant mice for biological functions. **(A–D)** GSEA results for W3-HT transcripts showing the list of top five positively (red) and negatively (blue) enriched gene sets **(A,C)** and their integrated visualization generated using EnrichmentMap Cytoscape App **(B,D)**. Only the results for C5-CC (cellular component) are shown here, and those for C5-BP (biological process) and C5-MF (molecular function) are shown in Supplementary Figures 2, 3. Note that, although the top five gene sets are shown in the main figure tables for simplicity (see the full lists in Supplementary Tables 4–7), EnrichmentMap visualization utilized all of the GSEA results (*n* = 5 mice for WT, HT, and HM, FDR < 0.05). **(E–H)** GSEA results for W3-HM transcripts **(E,G)** and their integrated visualization generated using EnrichmentMap **(F,H)** (*n* = 5 mice for WT, HT, and HM, FDR < 0.05). See Supplementary Figures 4, 5 for GSEA results for C5-BP and C5-MF.

Gene Set Enrichment Analysis of W3-HT transcripts indicated positive enrichment of the ranked genes (meaning that the upregulated genes are enriched) for synapse- or excitatory postsynapse-related gene sets in the CC, BP, and MF domains of the C5 gene sets (Gene Ontology gene sets; 10,185 gene sets as of now), as shown by the top five gene sets enriched [Figure 3A; Supplementary Figures 2, 3; and Supplementary Table 4 (CC/BP/MF-Up tabs)]. Integration of all of the GSEA results (not just the top five or 10) and the use of EnrichmentMap Cytoscape App (Merico et al., 2010) to visualize clusters of biological functions further highlighted the apparent importance of synapse-related functions and (to a lesser extent) chromatin- and cell body/axon-related functions (Figure 3B). W3-HT transcripts were negatively enriched for ribosome- and mitochondria-related gene sets in the C5-CC, C5-BP, and C5-MF domains, and these findings were supported by EnrichmentMap visualization [Figures 3C,D; Supplementary Figures 2, 3; and Supplementary Table 4 (CC/BP/MF-Down tabs)]. These functional associations were generally stronger in CC and BP domains relative to the MF domain.

Gene Set Enrichment Analysis of W3-HM transcripts indicated positive enrichment for chromatin- and RNA-related gene sets in the C5-CC, C5-BP, and C5-MF domains [Figures 3E,F; Supplementary Figures 4, 5; and Supplementary Table 5 (CC/BP/MF-Up tabs)]. In addition, W3-HM transcripts were, to a modest degree, negatively enriched for lipid/vacuole- and cation/anion transport-related gene sets [Figures 3G,H; Supplementary Figures 4, 5; and Supplementary Table 5 (CC/BP/MF-Down tabs)]. These results suggest that W3-HT (up and down) and W3-HM (up and down) transcripts are enriched in four distinct groups of genes.

**FIGURE 4:**
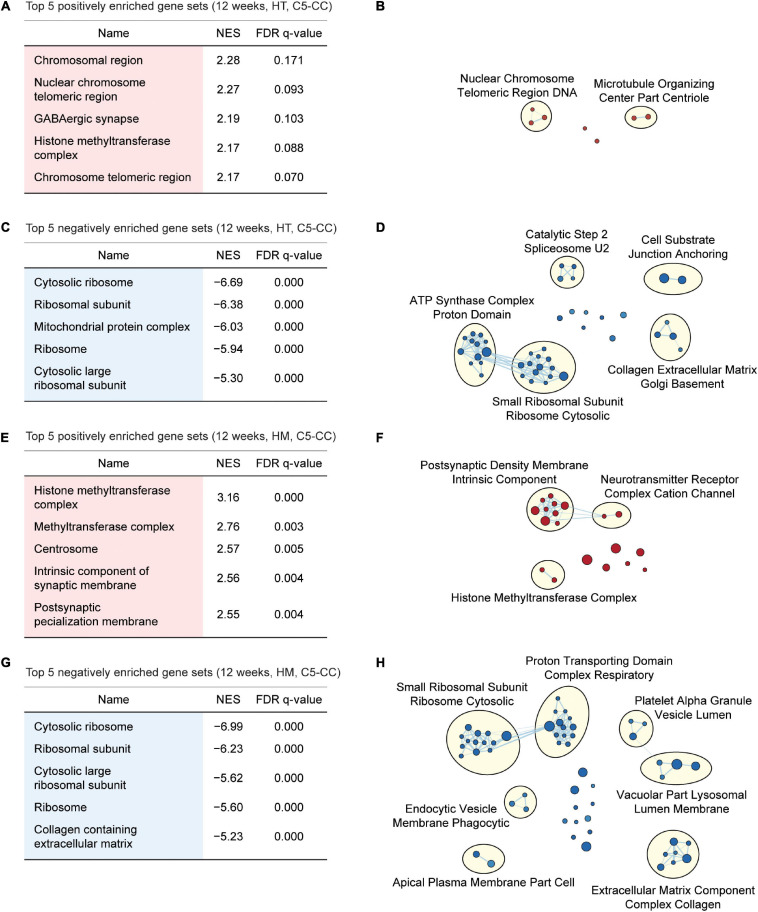
GSEA of transcriptomes from W12-HT and W12-HM *Shank2*-mutant mice for biological functions. **(A–D)** GSEA results for W12-HT transcripts showing the list of top five positively (red) and negatively (blue) enriched gene sets **(A,C)** and their integrated visualization using EnrichmentMap Cytoscape App **(B,D)**. See Supplementary Figures 6, 7 for GSEA results for C5-BP and C5-MF (*n* = 4 mice for WT, HT, and HM, FDR < 0.05). **(E–H)** GSEA results for W12-HM transcripts **(E,G)** and their integrated visualization generated using EnrichmentMap **(F,H)** (*n* = 4 mice for WT, HT, and HM, FDR < 0.05). See Supplementary Figures 8, 9 for GSEA results for C5-BP and C5-MF.

Gene Set Enrichment Analysis of W12-HT transcripts indicated positive but weak enrichment for synapse- and chromatin-related gene sets in the C5-CC, C5-BP, and C5-MF domains [Figures 4A,B; Supplementary Figures 6, 7; and Supplementary Table 6 (CC/BP/MF-Up tabs)], which contrasts with the strong synapse-related enrichments of W3-HT transcripts. W12-HT transcripts showed strong negative enrichments for ribosome- and mitochondria-related gene sets [Figures 4C,D; Supplementary Figures 6, 7; and Supplementary Table 6 (CC/BP/MF-Down tabs)], similar to the results from W3-HT transcripts.

W12-HM transcripts showed weakly positive enrichments for chromatin- and synapse-related gene sets [Figures 4E,F; Supplementary Figures 8, 9; and Supplementary Table 7 (CC/BP/MF-Up tabs)], similar to the results from W12-HT transcripts. In addition, W12-HM transcripts were negatively enriched for ribosome-related gene sets [Figures 4G,H; Supplementary Figures 8, 9; and Supplementary Table 7 (CC/BP/MF-Down tabs)], similar to the results from W3-HT (but not W3-HM) and W12-HT transcripts.

The abovementioned associations of the four transcript sets (W3-HT, W3-HM, W12-HT, and W12-HM) with key biological functions, including synapse, ribosome, mitochondria, and chromosome, were further highlighted by the list of specific genes that played key roles in the enrichments [Supplementary Table 8; a summary of key genes in the top gene set in each category (CC-up/down, BP-up/down, and MF-up/down domains of the four transcript sets) in the tables of Figures 3, 4]. For example, key upregulated synapse-related genes from W3-HT transcripts (CC domain) included Dmd (dystrophin), Slc8A1 (solute carrier family 8 member A1, a Na/Ca exchanger), Kcnb1 (potassium voltage-gated channel subfamily B member 1), Atp2b2 (ATPase plasma membrane Ca^2+^ transporting 2), and Grin2b (glutamate ionotropic receptor NMDA type subunit 2B). Key downregulated genes associated with ribosome/mitochondria-related functions from W3-HT transcripts (CC domain) included Rps27 (ribosomal protein S27), Malsu1 (mitochondrial assembly Of ribosomal large subunit 1), Larp4 (La ribonucleoprotein 4, an RNA-binding protein), Eif2d (eukaryotic translation initiation factor 2D), Nsun4 (NOP2/Sun RNA methyltransferase 4), and multiple Mrp (mitochondrial ribosomal protein) genes (i.e., MRPS6/MRPS7/MRPS5/MRPL22/MRPL4/MRPL1). Key upregulated chromatin-related genes from W3-HM transcripts included Nabp1 (nucleic acid binding protein 1), Cbx5 (Chromobox 5), Smarca1 (SWI/SNF related, matrix associated, actin dependent regulator of chromatin, subfamily A, member 1), Ago3 (Argonaute RISC catalytic component 3, with roles in RNA interference), Mef2c [myocyte enhancer factor 2C in the MADS box transcription enhancer factor 2 (MEF2) family].

### GSEA of Shank2-Mutant Transcriptomes for ASD-Related/Risk Gene Sets

Previous studies have reported transcriptomic alterations in ASD (Garbett et al., 2008; Voineagu et al., 2011; Gupta et al., 2014; Parikshak et al., 2016; Velmeshev et al., 2019) and gene sets that are distinctly up- or downregulated in ASD such as DEG_Up_Voineagu, Co-Exp_Up_M16_Voineagu, DEG_Down_Voineagu, and Co-Exp_Down_M12_Voineagu (cortical samples with the age range of 2–56) (Voineagu et al., 2011; Werling et al., 2016) (see Supplementary Table 9 for the whole list of genes). In addition, multiple genes sets for ASD-risk genes have been reported; SFARI genes (all) (Abrahams et al., 2013), SFARI (high confidence, belonging to category 1) (Abrahams et al., 2013) (see text footnote 5), FMRP targets (Darnell et al., 2011; Werling et al., 2016), DeNovoMis (protein-disrupting or missense rare *de novo* variants) (Iossifov et al., 2014; Werling et al., 2016), DeNovoVariants (protein-disrupting rare *de novo* variants) (Iossifov et al., 2014; Werling et al., 2016), and AutismKB (Autism KnowledgeBase) (Xu et al., 2012; Yang et al., 2018; Supplementary Table 9). These ASD-related/risk gene sets were also found to be enriched in the transcriptomes of mice carrying ASD-risk mutations such as *Chd8*- and *Tbr1*-mutant mice in ways similar to those observed in ASD (Jung et al., 2018; Yook et al., 2019). We thus tested whether the four transcript groups (W3-HT, W3-HM, W12-HT, and W12-HM; whole list of genes ranked by *p* values) from *Shank2*-mutant mice show enrichments for these ASD-related/risk gene sets using the same GSEA approach.

**FIGURE 5:**
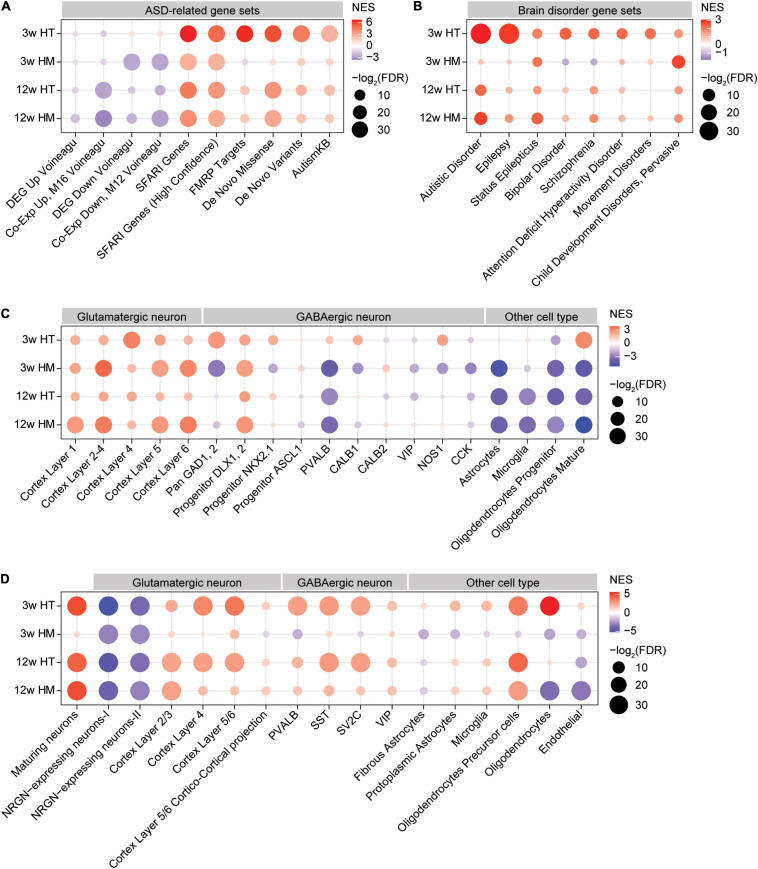
GSEA of transcriptomes from W3-HT, W3-HM, W12-HT, and W12-HM *Shank2*-mutant mice for ASD- and cell type-related gene sets. **(A,B)** GSEA results for all four transcript groups (W3-HT, W3-HM, W12-HT, and W12-HM) showing enrichment patterns for ASD- and brain disorder-related gene sets. The entire lists of genes with transcriptional changes ranked by *p* values were used as inputs. Note that the ASD-related gene sets include those that are upregulated in ASD (DEG_Up_ Voineagu and Co-Exp_Up_M16_ Voineagu), those that are downregulated in ASD (DEG_Down_Voineagu and Co-Exp_Down_M12_ Voineagu), and that ASD-risk genes are likely to be downregulated in ASD [SFARI (all), SFARI (high confidence), FMRP targets, DeNovoMis, DeNovoVariants, and AutismKB] (see Supplementary Table 9 for gene-set details) (*n* = 5 mice for W3-WT/HT/HM and four mice for W12-WT/HT/HM, FDR > 0.05). **(C)** GSEA results for all four transcript groups (W3-HT, W3-HM, W12-HT, and W12-HM) showing enrichment patterns for specific cell type-related gene sets (cell-type-specific; see Supplementary Table 9 for gene-set details). Note that ASD has been associated with downregulation of neuron (glutamate and GABA)- and oligodendrocyte-related genes and upregulation of glia (astrocyte and microglia)-related genes (*n* = 5 mice for W3-WT/HT/HM and four mice for W12-WT/HT/HM). **(D)** GSEA results for all four transcript groups (W3-HT, W3-HM, W12-HT, and W12-HM) showing enrichment patterns for cell-type-specific genes validated by single-cell RNA-Seq results of human cortical tissues (single-cell-type-specific; see Supplementary Table 9 for gene-set details) (*n* = 5 mice for W3-WT/HT/HM and four mice for W12-WT/HT/HM).

In W3-HT, the transcript pattern of ASD-related genes was strongly opposite that seen in ASD patients and model mice: W3-HT transcripts were negatively and moderately enriched for gene sets that are upregulated in ASD (DEG_Up_Voineagu and Co-Exp_Up_M16_Voineagu) and positively enriched for gene sets that are downregulated in ASD (DEG_Down_Voineagu and Co-Exp_Down_M12_Voineagu) (Figure 5A). Moreover, W3-HT transcripts were positively enriched for ASD-risk gene sets that are usually downregulated in ASD likely through missense, non-sense, splice-site, frame-shift, and deletion mutations; these included SFARI genes (all), SFARI (high confidence), FMRP targets, DeNovoMis, DeNovoVariants, and AutismKB (Figure 5A). Therefore, W3-HT transcripts show patterns that are strongly opposite to those observed in ASD (here, called “anti-ASD”).

W3-HM transcripts were negatively enriched for gene sets that are upregulated in ASD (DEG_Up_ Voineagu and Co-Exp_Up_M16_ Voineagu) (Figure 5A), similar to the pattern in W3-HT transcripts (anti-ASD). However, W3-HM transcripts were negatively and strongly enriched for gene sets downregulated in ASD (DEG_Down_Voineagu and Co-Exp_Down_M12_ Voineagu) (Figure 5A), and thus also exhibited some “pro-ASD” transcription. W3-HM transcripts were positively enriched for SFARI genes (all), SFARI genes (high confidence), DeNovoMis, DeNovoVariants, and AutismKB but negatively enriched for FMRP targets (Figure 5A). These results from W3-HM mice partly deviate from the W3-HT pattern and suggest that an increase in the dosage of *Shank2* deletion dampens the anti-ASD transcriptomic changes at W3.

W12-HT and W12-HM transcripts showed patterns in line with anti-ASD changes. The W12-HT/HM transcripts were negatively enriched for gene sets upregulated in ASD (DEG_Up_ Voineagu and Co-Exp_Up_M16_ Voineagu), although they were negatively enriched for some gene sets downregulated in ASD (DEG_Down_Voineagu and Co-Exp_Down_M12_ Voineagu). W12-HT/HM transcripts were also positively enriched for SFARI genes (all), SFARI (high confidence), FMRP targets, DeNovoMis, DeNovoVariants, and AutismKB (Figure 5A). These patterns are more similar to that of W3-HM than W3-HT.

The four transcript groups (W3-HT, W3-HM, W12-HT, and W12-HM) were minimally enriched for other brain disorder-related gene sets, compared with ASD-related/risk gene sets, in DisGeNet^5^ (Pinero et al., 2020; Figure 5B). One exception was the relatively strong enrichment of W3-HT, but not the other three transcripts, for genes related to epilepsy, in line with that (1) epilepsy is one of the comorbidities of ASD, (2) epilepsy symptoms were observed in individuals with *SHANK2*-related genetic variations (although rare) (Leblond et al., 2012), and (3) *Shank2* expression is altered in epileptic humans and rat models (Fu et al., 2020). These results collectively suggest that W3-HT transcripts show unique and strong anti-ASD transcriptomic changes, whereas the other three transcript sets (W3-HM, W12-HT, and W12-HM) show both anti-ASD and pro-ASD changes.

### GSEA of Shank2-Mutant Transcriptomes for Cell Type-Specific Gene Sets

Autism spectrum disorders has been associated with cell-type-specific changes in gene expression, including decreased neuronal gene expression and increased/decreased glial cell expression (increased astrocyte/microglia-related genes and decreased oligodendrocyte-related genes) (Voineagu et al., 2011; Werling et al., 2016). We thus tested if Shank2 transcripts from W3/12-HT/HM mice show any cell-type-specific enrichments, using cell-type-specific gene sets (Kang et al., 2011) and single-nucleus cell-type-specific gene sets (Velmeshev et al., 2019) (see Supplementary Table 9 for further details).

W3-HT transcripts were positively enriched for cortical glutamate neuron-related gene sets identified across different cortical layers (Figure 5C; Kang et al., 2011). Similarly, W3-HT transcripts were largely positively enriched for GABA neuron-related gene sets, including those for parvalbumin-positive neurons (Figure 5C; Kang et al., 2011). For glia-related gene sets, W3-HT transcripts were positively and strongly enriched for oligodendrocyte (mature)-, but not for astrocyte- or microglia-related gene sets.

W3-HM transcripts were also positively enriched for cortical glutamate neuron-related gene sets, similar to W3-HT transcripts; however, they were largely negatively enriched for GABA neuron-related gene sets, including those associated with parvalbumin-positive neurons (Figure 5C). W3-HM transcripts were negatively enriched for all three glia-related gene sets (astrocyte, microglia, and oligodendrocytes).

W12-HT and W12-HM transcripts were positively enriched for cortical glutamate neuron-related gene sets and largely negatively enriched for GABA neuron-related gene sets (Figure 5C). In addition, W12-HT and W12-HM transcripts were negatively enriched for glia-related gene sets (astrocytes, microglia, and oligodendrocytes).

Therefore, W3-HT transcripts are strongly anti-ASD based on upregulated neuronal (both glutamate and GABA) and oligodendrocyte genes, whereas the other three transcripts (W3-HM, W12-HT, and W12-HM) are partly anti-ASD (upregulated glutamate neuronal genes and downregulated astrocyte/microglia genes) and pro-ASD (downregulated oligodendrocyte genes).

Lastly, the four transcript groups (W3-HT, W3-HM, W12-HT, and W12-HM) were tested cell-type-specific genes validated by single-cell RNA-Seq results of human cortical tissues (Velmeshev et al., 2019). The overall enrichment patterns were similar to those described above (Figure 5C) in that W3-HT transcripts were uniquely and strongly anti-ASD, with upregulated neuronal and oligodendrocyte genes, and that the other three transcripts were less strongly anti-ASD, as shown by moderately upregulated neuronal and oligodendrocyte genes and largely downregulated astrocyte/microglia genes (Figure 5D).

## Discussion

In the present study, we analyzed and compared the transcriptomic changes occurring in the PFC of four different Shank2-mutant mouse groups (W3-HT, W3-HM, W12-HT, and W12-HM) using DEG and GSEA analyses. Our goal was to provide insight into the molecular changes induced in the PFC by *Shank2* heterozygous and homozygous deletions across juvenile and adult stages in mice.

Our analyses identified differential transcriptomic changes in heterozygous and homozygous *Shank2*-mutant mice across juvenile and adult stages and interesting biological functions, including those associated with synapse, ribosome, mitochondria, chromatin, GABA neuron, and oligodendrocyte. Before interpreting these results, the following limitations of the study should be pointed out. First, the fact that some biological functions are missing in a subset of the four transcript groups does not necessarily mean that they do not exist. They could be present, but the statistical powers may be low; we used only 4–5 mice per group. Second, the biological functions identified in the present study involving up- or downregulations of the genes are those that work only at the transcript level and are not supported by any functional results. In addition, the transcriptional changes may not reflect functional up- or downregulations but merely reflect compensatory transcriptional changes, which often move toward opposite directions, although additional hints could come from whether the changes are anti- or pro-ASD (see below).

One interesting transcriptomic change was the increase in synapse (particularly excitatory postsynapse)-related gene expression in W3-HT mice, which was not observed in W3-HM, W12-HT, or W12-HM mice (Figures 3, 4). Given that Shank2 is a core component of excitatory postsynaptic protein complexes (Kim and Sheng, 2004; Grabrucker et al., 2011; Sala et al., 2015; Mossa et al., 2017), and the loss of *Shank2* exons 6 and 7 suppresses excitatory synaptic transmission mediated by NMDARs in the mPFC, hippocampus, and amygdala (Won et al., 2012; Lee et al., 2015; Chung et al., 2019), and that the W3-HT transcriptome has anti-ASD nature, the upregulation of excitatory synapse-related genes in W3-HT mice is likely to represent indirect changes that compensate for reduced excitatory transmission. However, although W3-HM mice show decreased NMDAR function in the mPFC, the previous studies did not directly test if W3-HT mice show reduced excitatory synaptic transmission in the PFC or hippocampus (Won et al., 2012; Chung et al., 2019).

Although W3-HM mice did not display altered synapse-related gene expression, they did show increased chromatin-related gene expression. This suggests that a stronger *Shank2* deletion (HM relative to HT) converts the “color” of the transcriptomic changes from synapse- to chromatin-related gene expression changes. It is tempting to speculate that broader changes in the expression levels of chromatin remodeling-related genes are required to compensate for the deficits induced by a stronger *Shank2* deletion. In line with this, human cortical neurons carrying *SHANK2* mutations with excitatory synaptic hyperconnectivity show chromatin-related changes in gene expression (Zaslavsky et al., 2019). In addition, chromatin-related changes have frequently been associated with ASD (De Rubeis et al., 2014) and observed in mice lacking *Shank3* (a relative of *Shank2*) (Zhu et al., 2014; Ma et al., 2018; Qin et al., 2018; Wang et al., 2019; Zhang et al., 2021).

The increased synaptic gene expression observed in W3-HT mice is markedly weakened in W12-HT mice, where only moderate increases in synapse- and chromatin-related gene expression were observed. It is possible that the continued absence of *Shank2* expression across juvenile and adult stages dampens the compensatory gene expression changes. In addition, it could be that the period of active synapse development and flexible synapse-related transcriptomic changes comes to an end roughly around the third postnatal week and does not persist into adulthood (Sheng and Kim, 2011). Moreover, the prolonged heterozygous *Shank2* deletion might have changed the nature of the compensatory responses from synapse-only changes to those involving both synapse- and chromatin-related gene expressions. Notably, W12-HM mice show transcript patterns that are similar to those of W12-HT mice (moderate increases in synapse- and chromatin-related gene expressions), suggesting that prolonged *Shank2* deletion weakens the dosage-dependent effects of *Shank2* loss at W12, which were strong at W3 between HT and HM mice. This could be attributable to age-dependent changes or some compensatory changes such as altered expression of other members of the Shank family (i.e., Shank3), as reported previously (Schmeisser et al., 2012).

Another notable transcriptomic change observed in W3-HT mice was the downregulations of ribosome- and mitochondria-related genes. This change was not observed in W3-HM mice, where only moderate decreases in lipid- and cation transport-related gene expression levels were observed. This suggests that *Shank2* deletions of different dosages induce distinct changes in gene downregulations at W3, similar to the distinct upregulatory patterns seen at W3 (synapse- and chromatin-related genes in W3-HT and W3-HM mice, respectively). Notably, equally strong decreases in ribosome/mitochondria-related gene expression levels were observed in W12-HT mice, suggesting that, unlike the substantial dampening of synapse gene upregulations across W3 and W12, there is no age-dependent dampening of transcriptomic downregulations. In addition, W12-HM mice also showed strong decreases in ribosome-related gene expressions. It thus seems that decreases in ribosome/mitochondria-related gene expression levels represent the strongest and most widespread transcriptomic changes observed in three out of the four transcript sets (W3-HT, W12-HT, and W12-HM, but not W3-HM). Although we named some of the genes associated with ribosome/mitochondrial functions in Results based on the relative contributions to the enrichments, caution should be taken in narrowing down to specific genes because there are multiple gene sets associated with a single biological function (i.e., ribosome), and there are multiple genes in a single gene set that contributed to the enrichments. It is possible that moderate but coordinated transcriptional changes in many genes can lead to strong gene-set enrichments, which might be occurring in ribosome/mitochondria genes in Shank2-mutant mice.

It is unclear how ribosome/translation-related genes are downregulated in *Shank2*-mutant mice. Protein synthesis is thought to be reciprocally connected with synaptic proteins and functions under the context of ASD-risk gene functions (Santini and Klann, 2014). In addition, Shank2 is known to coordinate excitatory synaptic signaling pathways (Grabrucker et al., 2011; Sheng and Kim, 2011; Sala et al., 2015; Mossa et al., 2017). It is, therefore, possible that suppressed NMDAR or excitatory synaptic functions in *Shank2*-mutant mice are associated with the decreased ribosome/translation-related transcriptomic activities, perhaps through mTOR signaling, a central regulator of translational initiation and synaptic and neuronal functions implicated in various brain disorders, including ASD (Hoeffer and Klann, 2010; Costa-Mattioli and Monteggia, 2013; Borrie et al., 2017; Saxton and Sabatini, 2017; Switon et al., 2017; Winden et al., 2018; Bagni and Zukin, 2019; Sossin and Costa-Mattioli, 2019) and known to be regulated by synaptic receptors, including NMDARs and metabotropic glutamate receptors (mGluRs) (Hoeffer and Klann, 2010). However, our GSEA on *Shank2*-mutant transcriptomes did not detect any significant changes in mTOR signaling-related gene expressions, although altered mTOR signaling should be directly tested at the protein level using protein phosphorylation/activity as readouts. Intriguingly, bi-allelic but not mono-allelic deletion of *Shank2* has been shown to increase Akt phosphorylation without changes in total protein levels during early neuronal differentiation in SH-SY5Y cells, which involved decreased apoptosis and increased cell proliferation (Unsicker et al., 2021), suggesting that Shank2 regulates mTOR signaling downstream of receptor tyrosine kinase activation.

Intriguingly, mitochondria-related gene expressions are changed largely in parallel with those of ribosome/translation-related genes in W3-HT, W12-HT, and W12-HM mice. Mitochondrial dysfunctions have been extensively implicated in ASD (Lombard, 1998; Oliveira et al., 2005; Weissman et al., 2008; Giulivi et al., 2010; Frye and Rossignol, 2011; Anitha et al., 2012; Rossignol and Frye, 2012; Frye et al., 2013; Legido et al., 2013; Goh et al., 2014; Siddiqui et al., 2016; Hollis et al., 2017; Khemakhem et al., 2017; El-Ansary et al., 2018; Rose et al., 2018; Frye, 2020; Singh et al., 2020). In addition, mitochondria critically regulate synapse formation and synaptic transmission and plasticity (Li et al., 2004, 2020; Vos et al., 2010; Sheng and Cai, 2012; Lee A. et al., 2018; Rossi and Pekkurnaz, 2019). It is possible that excitatory synaptic defects induced by *Shank2* deletion, likely accompanying altered mTOR and ribosome/translation functions, may affect mitochondrial functions. Indeed, mTOR signaling regulates mitochondrial functions, in addition to protein synthesis, in mammalian cells to coordinate the energy consumption involved in protein translation with the energy production by mitochondria (Morita et al., 2015). Alternatively, mitochondria carry a few own genes required for oxidative phosphorylation, and mitochondrial ribosomes (mitoribosomes) are required for their translation (Amunts et al., 2015; Greber et al., 2015). Notably, downregulated ribosome-related genes from W3-HM transcripts included those for multiple MRPs (nuclear-encoded mitochondrial ribosomal, or mitoribosomal, proteins), known to regulate mitoribosome biogenesis and mitochondrial translation implicated in human diseases (Smits et al., 2010; Boczonadi and Horvath, 2014), and Malsu1, a regulator of mitoribosome biogenesis and mitochondrial translation (Rorbach et al., 2012; Wanschers et al., 2012; Fung et al., 2013). It is possible that mitochondrial dysfunctions in *Shank2*-mutant mice might induce downregulations of genes associated with mitoribosome biogenesis and mitochondrial translation.

GSEA for ASD-related gene sets indicate that W3-HT transcripts show enrichment patterns that are strongly opposite to those observed in ASD (Figure 5). The gene sets that are downregulated in ASD are negatively enriched (pro-ASD) in W3-HM, W12-HT, and W12-HM, but positively enriched in W3-HT (anti-ASD). In addition, the positive enrichment of W3-HT transcripts for synapse-related gene sets is opposite to the finding that synapse-related gene functions are downregulated in ASD post-mortem cortex (Voineagu et al., 2011). In these contexts, therefore, W3-HT transcripts again show the strongest and most unique anti-ASD patterns.

The four transcript groups are also similarly enriched for the ASD-related cell-type gene sets. W3-HT transcripts show positive enrichment for glutamate neuron-related gene sets (anti-ASD), and similar results are seen in the other three groups (W3-HM, W12-HT, and W12-HM). However, W3-HT transcripts are positively enriched for GABA neuron-related gene sets (anti-ASD), whereas the other three groups show negative enrichments, or less strong positive enrichments, for GABA neuronal genes (pro-ASD). Given the decreased neuronal gene expression in ASD (Voineagu et al., 2011) and mouse models of ASD (Jung et al., 2018; Yook et al., 2019), these results, together with the abovementioned results, indicate that W3-HT transcripts are uniquely and strongly anti-ASD. These early and strong transcriptomic changes might underlie the lack of strong autistic-like behavioral phenotypes in heterozygous *Shank2*-mutant mice, although hyperactivity was observed in these mice (Won et al., 2012). In addition, these results further suggest that decreased GABA neuron-related gene expression and consequent GABA neuronal dysfunction might promote ASD-like phenotypes in *Shank2*-mutant mice. Consistent with this possibility, parvalbumin-positive neuron-specific deletion of *Shank2* (exon 6 + 7) leads to moderate hyperactivity, enhanced self-grooming, and suppressed seizure susceptibility in mice (Lee S. et al., 2018). In addition, promoting GABAergic synaptic transmission rescues cognitive deficits observed in adult homozygous *Shank2*-mutant mice (Lim et al., 2017).

W3-HT transcripts show strong positive enrichment for oligodendrocyte-related gene sets, whereas the other three groups show negative, or only moderate, enrichments. Given that downregulation of oligodendrocyte-related gene sets, but not those related to astrocytes or microglia, has been implicated in ASD (Voineagu et al., 2011), our present results suggest that W3-HT transcripts but not the other three are anti-ASD patterns. Whether this difference differentially affects the density and function of oligodendrocytes in *Shank2*-mutant mice remains to be determined, although abnormalities in oligodendrocytes and axonal myelination have been associated with ASD (Graciarena et al., 2018; Galvez-Contreras et al., 2020; Kawamura et al., 2020a,b).

In summary, our study demonstrates that heterozygous and homozygous *Shank2* (exons 6 + 7) deletions in mice at W3 and W12 induce differential transcriptomic changes, and that these are distinct at W3 but become more similar at W12. Our study also identifies distinct functional groups of genes whose expression changes are associated with *Shank2* deletions in mice, such as those related to synapses, chromatin, ribosomes, mitochondria, GABA neurons, and oligodendrocytes.

## Data Availability Statement

The RNA-Seq datasets presented in this study can be found in the GEO (Gene Expression Omnibus) repository under Accession Number (GSE168211).

## Ethics Statement

The animal study was reviewed and approved by the committee of animal research at KAIST (KA2020-99).

## Author Contributions

SL, EL, HJ, and HK performed RNA-Seq analysis. EK and EL designed research and wrote the manuscript. All authors contributed to the article and approved the submitted version.

## Conflict of Interest

The authors declare that the research was conducted in the absence of any commercial or financial relationships that could be construed as a potential conflict of interest.

## Footnotes

1 http://www.informatics.jax.org/homology.shtml

2 http://software.broadinstitute.org/gsea

3 http://software.broadinstitute.org/gsea/msigdb

4 https://gene.sfari.org/

5 https://www.disgenet.org/

## Supplementary Material

Supplementary Figure 1 Principle component analysis (PCA) and scatter plots for W3-WT, W3-HT, W3-HM, W12-WT, W12-HT, and W12-HM transcriptomes. **(A,B)** PCA plots for WT, HT, and HM transcriptomes at W3 **(A)** and W12 **(B)**. **(C,D)** Scatter plots for WT, HT, and HM transcriptomes combined at W3 **(C)** and W12 **(D)**, for independent quality control of the sequencing results, showing the percentage of mapped reads plotted against the number of mapped reads, which ranged 25–36 million and ∼80%.

Supplementary Figure 2 GSEA of transcriptomes from W3-HT *Shank2*-mutant mice for biological functions in the C5-BP (biological process) domain. **(A,B)** GSEA results for W3-HT transcripts showing the list of top five positively (red) and negatively (blue) enriched gene sets **(A)** and their integrated visualization generated using Cytoscape EnrichmentMap App **(B)** (*n* = 5 mice for WT, HT, and HM, FDR < 0.05).

Supplementary Figure 3 GSEA of transcriptomes from W3-HT *Shank2*-mutant mice for biological functions in the C5-MF (molecular function) domain. **(A,B)** GSEA results for W3-HT transcripts showing the list of top five positively (red) and negatively (blue) enriched gene sets **(A)** and their integrated visualization generated using Cytoscape EnrichmentMap App **(B)** (*n* = 5 mice for WT, HT, and HM, FDR < 0.05).

Supplementary Figure 4 GSEA of transcriptomes from W3-HM *Shank2*-mutant mice for biological functions in the C5-BP (biological process) domain. **(A,B)** GSEA results for W3-HM transcripts **(A)** and their integrated visualization generated using Cytoscape EnrichmentMap App **(B)** (*n* = 5 mice for WT, HT, and HM, FDR < 0.05).

Supplementary Figure 5 GSEA of transcriptomes from W3-HM *Shank2*-mutant mice for biological functions in the C5-MF (molecular function) domain. **(A,B)** GSEA results for W3-HM transcripts **(A)** and their integrated visualization generated using Cytoscape EnrichmentMap App **(B)** (*n* = 5 mice for WT, HT, and HM, FDR < 0.05).

Supplementary Figure 6 GSEA of transcriptomes from W12-HT *Shank2*-mutant mice for biological functions in the C5-BP (biological process) domain. **(A,B)** GSEA results for W12-HT transcripts showing the list of top five positively (red) and negatively (blue) enriched gene sets **(A)** and their integrated visualization generated using Cytoscape EnrichmentMap App **(B)** (*n* = 4 mice for WT, HT, and HM, FDR < 0.05).

Supplementary Figure 7 GSEA of transcriptomes from W12-HT *Shank2*-mutant mice for biological functions in the C5-MF (molecular function) domain. **(A,B)** GSEA results for W12-HT transcripts showing the list of top five positively (red) and negatively (blue) enriched gene sets **(A)** and their integrated visualization generated using Cytoscape EnrichmentMap App **(B)** (*n* = 4 mice for WT, HT, and HM, FDR < 0.05).

Supplementary Figure 8 GSEA of transcriptomes from W12-HM *Shank2*-mutant mice for biological functions in the C5-BP (biological process) domain. **(A,B)** GSEA results for W12-HM transcripts **(A)** and their integrated visualization generated using Cytoscape EnrichmentMap App **(B)** (*n* = 4 mice for WT, HT, and HM, FDR < 0.05).

Supplementary Figure 9 GSEA of transcriptomes from W12-HM *Shank2*-mutant mice for biological functions in the C5-MF (molecular function) domain. **(A,B)** GSEA results for W12-HM transcripts **(A)** and their integrated visualization generated using Cytoscape EnrichmentMap App **(B)** (*n* = 4 mice for WT, HT, and HM, FDR < 0.05).

Supplementary Table 1 Whole RNA-Seq results for the prefrontal cortex of W3-HT, W3-HM, W12-HT, and W12-HM *Shank2*-mutant mice.

Supplementary Table 2 List of upregulated and downregulated DEGs in W3-HT, W3-HM, W12-HT, and W12-HM *Shank2*-mutant mice.

Supplementary Table 3 DAVID analysis results (CC, BP, MF, and KEGG domains) for DEGs in W12-HT and W12-HM *Shank2*-mutant transcripts.

Supplementary Table 4 GSEA results (C5-CC, C5-BP, and C5-MF domains) for W3-HT *Shank2*-mutant transcripts.

Supplementary Table 5 GSEA results (C5-CC, C5-BP, and C5-MF domains) for W3-HM *Shank2*-mutant transcripts.

Supplementary Table 6 GSEA results (C5-CC, C5-BP, and C5-MF domains) for W12-HT *Shank2*-mutant transcripts.

Supplementary Table 7 GSEA results (C5-CC, C5-BP, and C5-MF domains) for W12-HM *Shank2*-mutant transcripts.

Supplementary Table 8 Details on the top enriched gene sets in C5 subdomains (CC, BP, and MF) for W3-HT, W3-HM, W12-HT, and W12-HM transcript groups, showing information on individual genes contributed to the enrichments such as original rank and running enrichment score.

Supplementary Table 9 ASD-related/risk and cell-type-specific gene sets used for GSEA of *Shank2*-mutant transcript groups (W3-HT, W3-HM, W12-HT, and W12-HM).

## Abbreviations

ASD: autism spectrum disorders
DEG: differentially expressed genes
GO: gene ontology
GSEA: gene set enrichment analysis
CC: cellular component
BP: biological process
MF: molecular function
SFARI: Simons Foundation Autism Research Initiative
AutismKB: Autism KnowledgeBase
KEGG: Kyoto Encyclopedia of Genes and Genomes
GABA: gamma amino butyric acid
