# Supplementary figures and images for: Gene Dosage- and Age-Dependent Differential Transcriptomic Changes in the Prefrontal Cortex of Shank2-Mutant Mice
Source: Front Mol Neurosci. 2021 Jun 11;14:683196. doi: 10.3389/fnmol.2021.683196 (PMC8226033; doi:10.3389/fnmol.2021.683196)

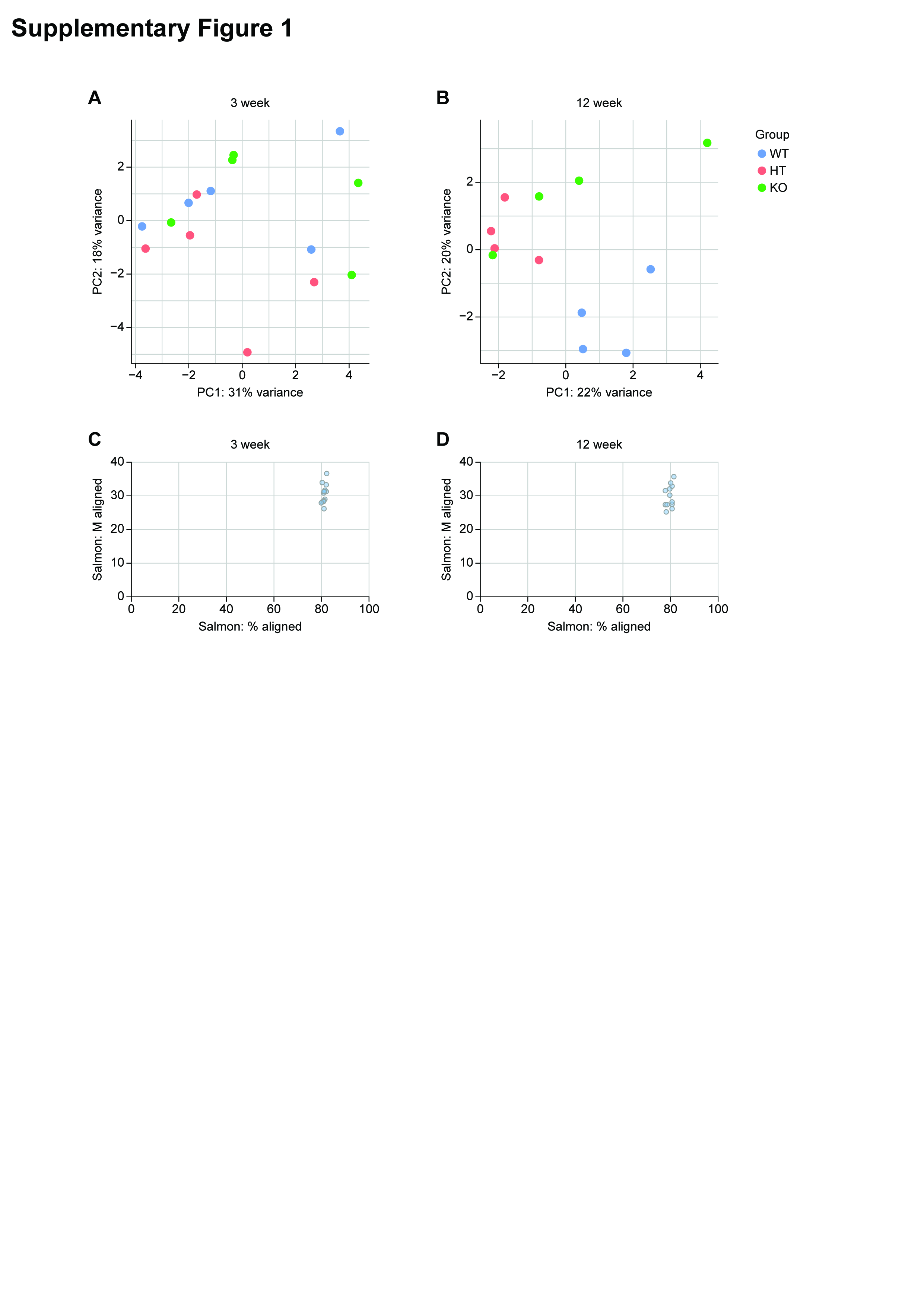

Supplement: Supplementary Figure 1 — Principle component analysis (PCA) and scatter plots for W3-WT, W3-HT, W3-HM, W12-WT, W12-HT, and W12-HM transcriptomes. (A,B) PCA plots for WT, HT, and HM transcriptomes at W3 (A) and W12 (B). (C,D) Scatter plots for WT, HT, and HM transcriptomes combined at W3 (C) and W12 (D), for independent quality control of the sequencing results, showing the percentage of mapped reads plotted against the number of mapped reads, which ranged 25–36 million and ∼80%. [file Image_1.TIF]

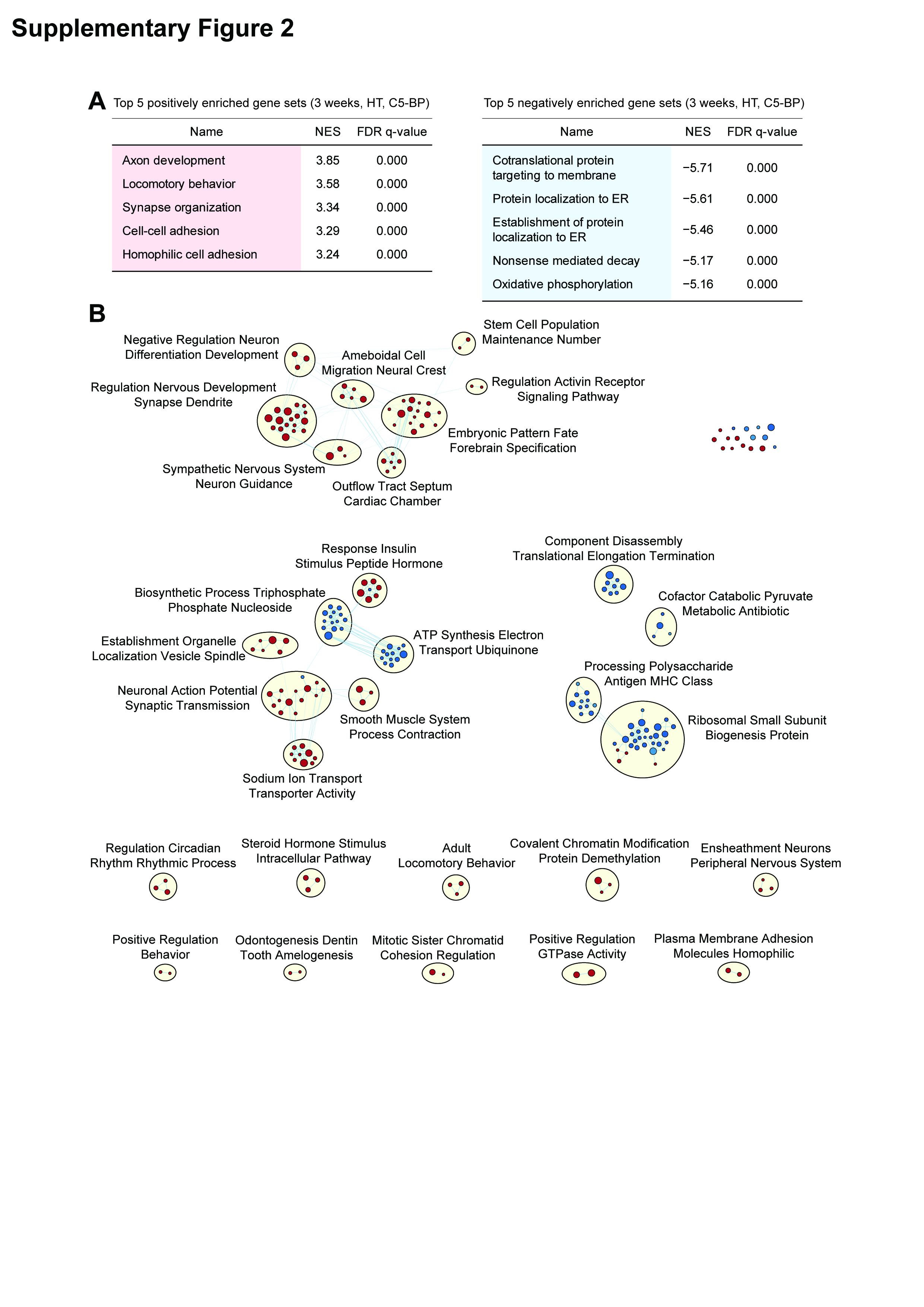

Supplement: Supplementary Figure 2 — GSEA of transcriptomes from W3-HT Shank2-mutant mice for biological functions in the C5-BP (biological process) domain. (A,B) GSEA results for W3-HT transcripts showing the list of top five positively (red) and negatively (blue) enriched gene sets (A) and their integrated visualization generated using Cytoscape EnrichmentMap App (B) (n = 5 mice for WT, HT, and HM, FDR < 0.05). [file Image_2.TIF]

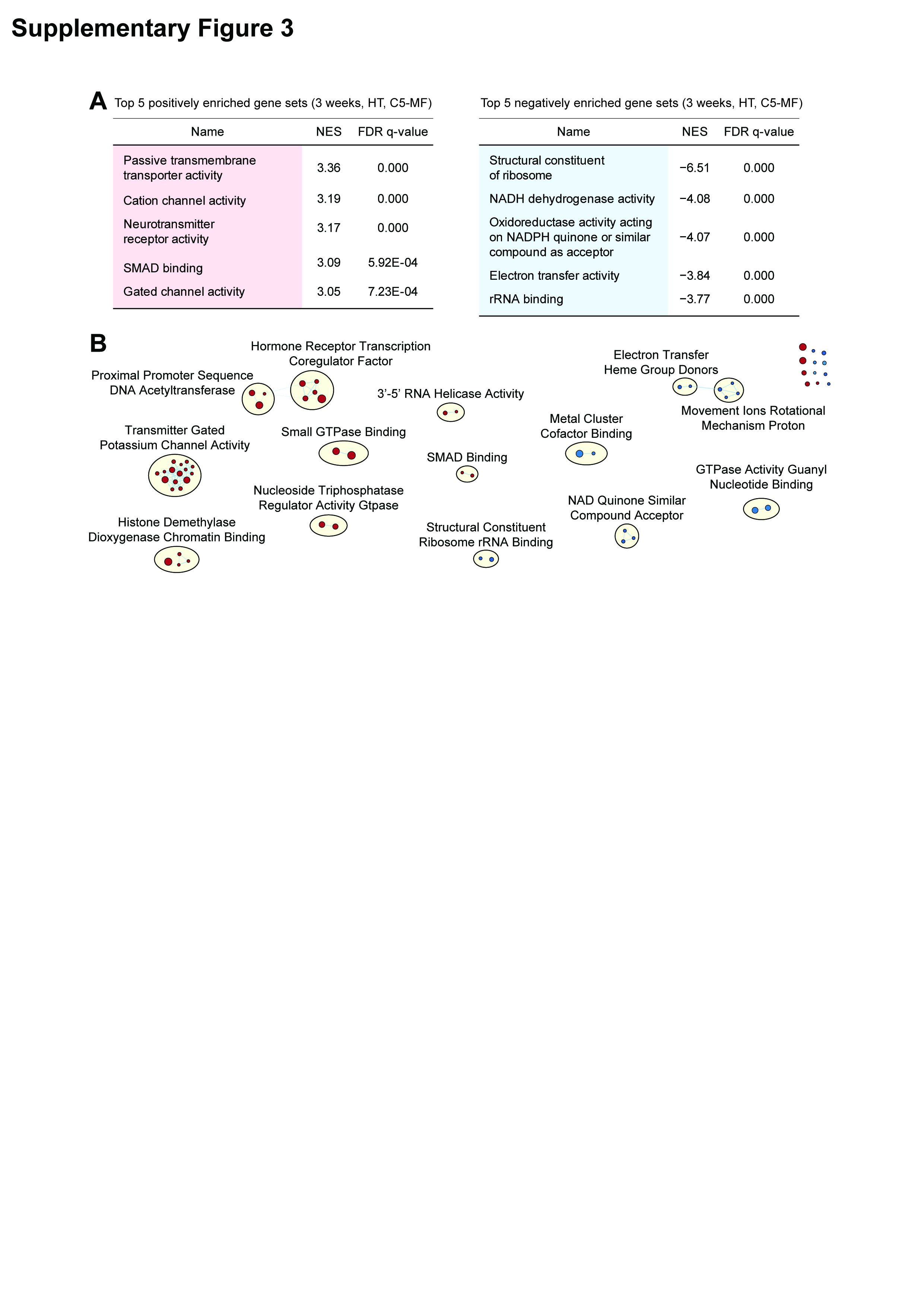

Supplement: Supplementary Figure 3 — GSEA of transcriptomes from W3-HT Shank2-mutant mice for biological functions in the C5-MF (molecular function) domain. (A,B) GSEA results for W3-HT transcripts showing the list of top five positively (red) and negatively (blue) enriched gene sets (A) and their integrated visualization generated using Cytoscape EnrichmentMap App (B) (n = 5 mice for WT, HT, and HM, FDR < 0.05). [file Image_3.TIF]

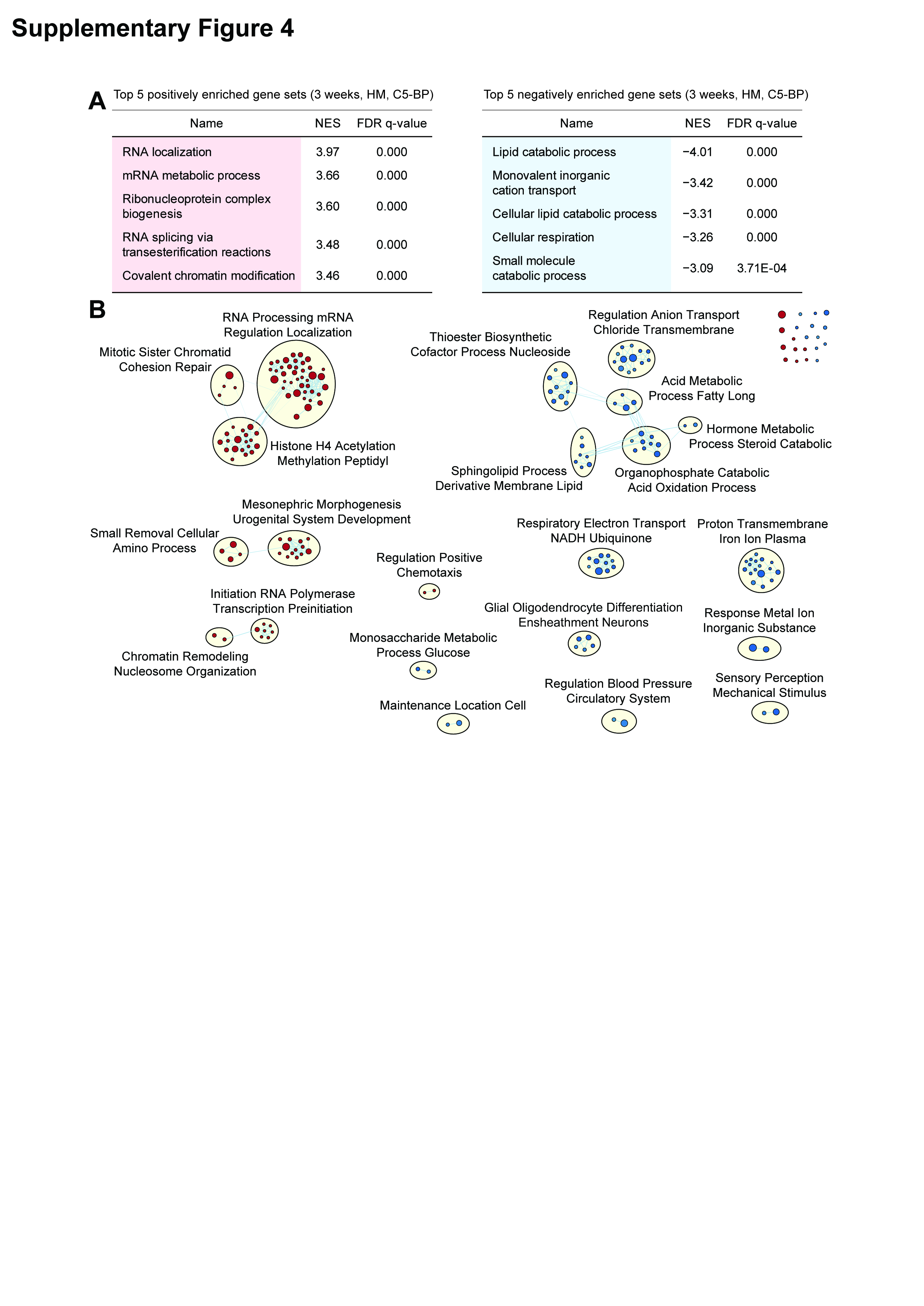

Supplement: Supplementary Figure 4 — GSEA of transcriptomes from W3-HM Shank2-mutant mice for biological functions in the C5-BP (biological process) domain. (A,B) GSEA results for W3-HM transcripts (A) and their integrated visualization generated using Cytoscape EnrichmentMap App (B) (n = 5 mice for WT, HT, and HM, FDR < 0.05). [file Image_4.TIF]

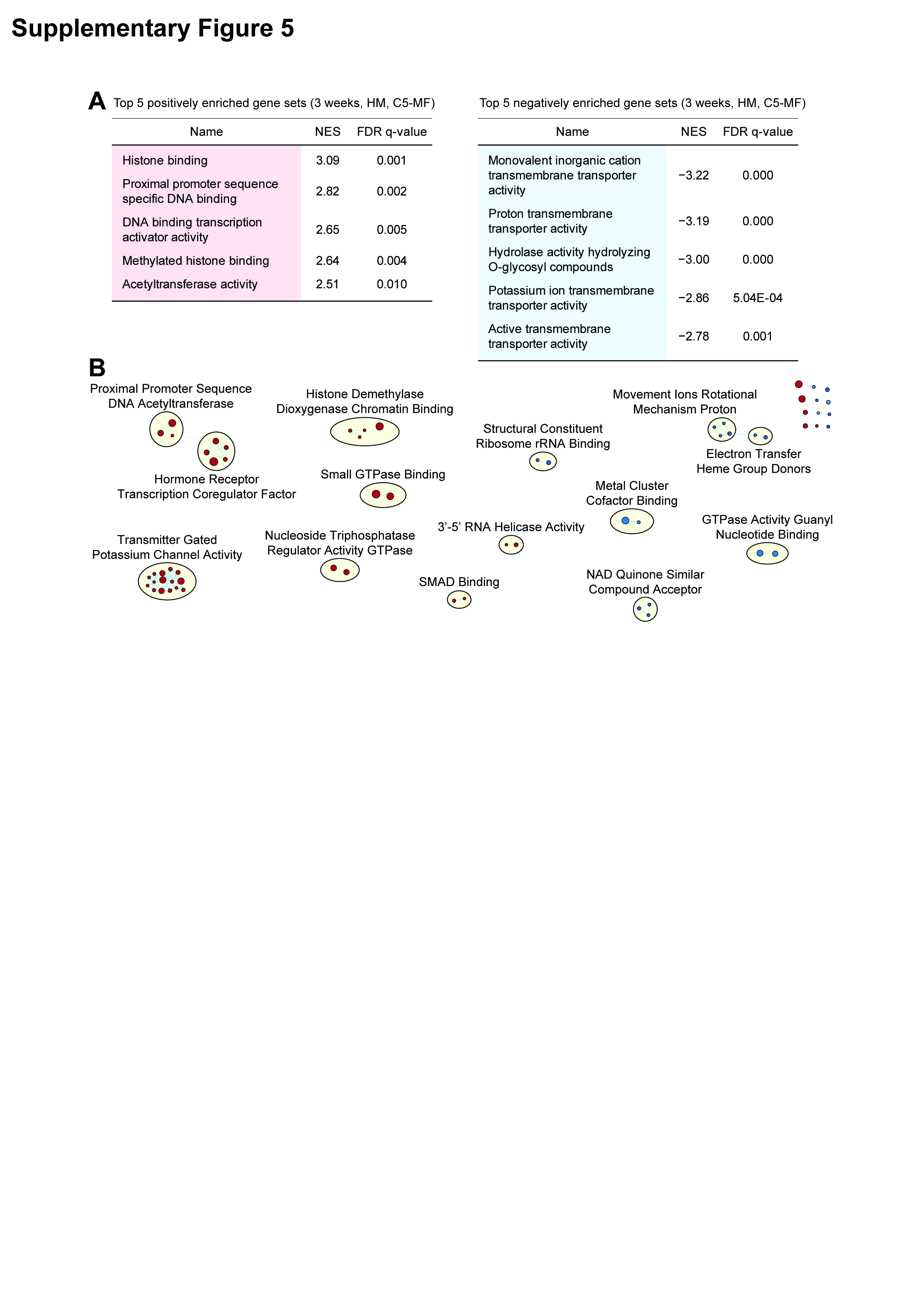

Supplement: Supplementary Figure 5 — GSEA of transcriptomes from W3-HM Shank2-mutant mice for biological functions in the C5-MF (molecular function) domain. (A,B) GSEA results for W3-HM transcripts (A) and their integrated visualization generated using Cytoscape EnrichmentMap App (B) (n = 5 mice for WT, HT, and HM, FDR < 0.05). [file Image_5.TIF]

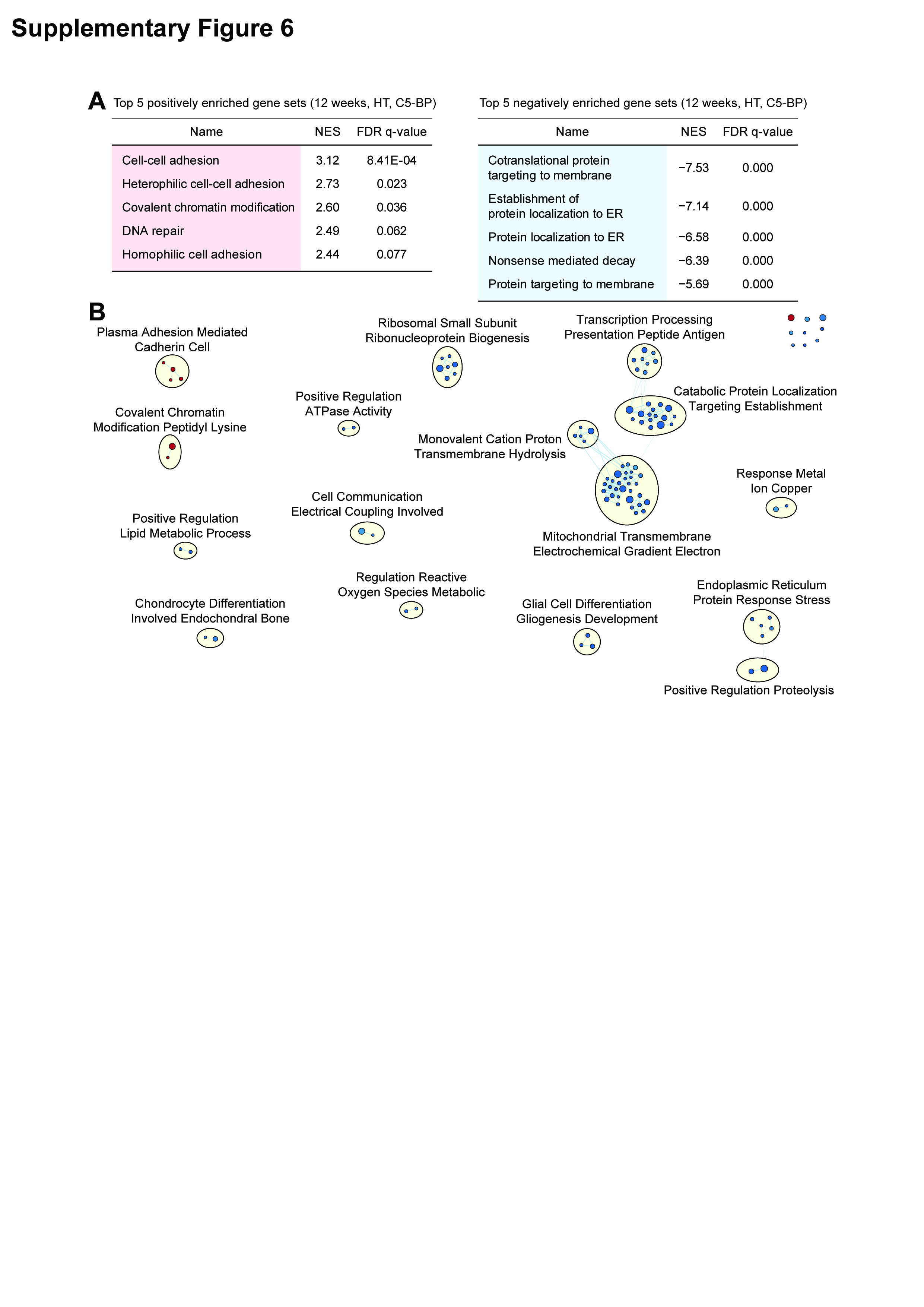

Supplement: Supplementary Figure 6 — GSEA of transcriptomes from W12-HT Shank2-mutant mice for biological functions in the C5-BP (biological process) domain. (A,B) GSEA results for W12-HT transcripts showing the list of top five positively (red) and negatively (blue) enriched gene sets (A) and their integrated visualization generated using Cytoscape EnrichmentMap App (B) (n = 4 mice for WT, HT, and HM, FDR < 0.05). [file Image_6.TIF]

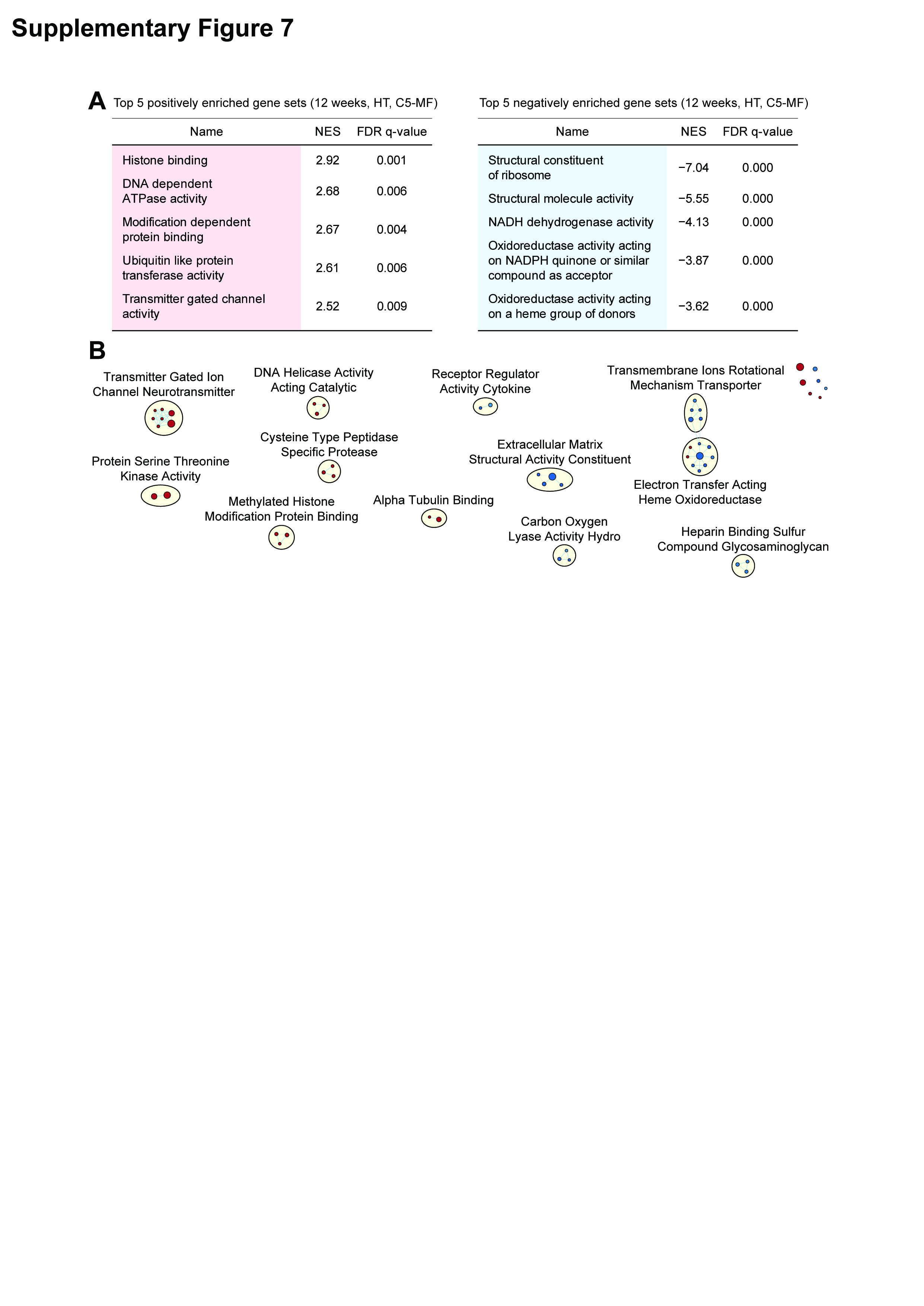

Supplement: Supplementary Figure 7 — GSEA of transcriptomes from W12-HT Shank2-mutant mice for biological functions in the C5-MF (molecular function) domain. (A,B) GSEA results for W12-HT transcripts showing the list of top five positively (red) and negatively (blue) enriched gene sets (A) and their integrated visualization generated using Cytoscape EnrichmentMap App (B) (n = 4 mice for WT, HT, and HM, FDR < 0.05). [file Image_7.TIF]

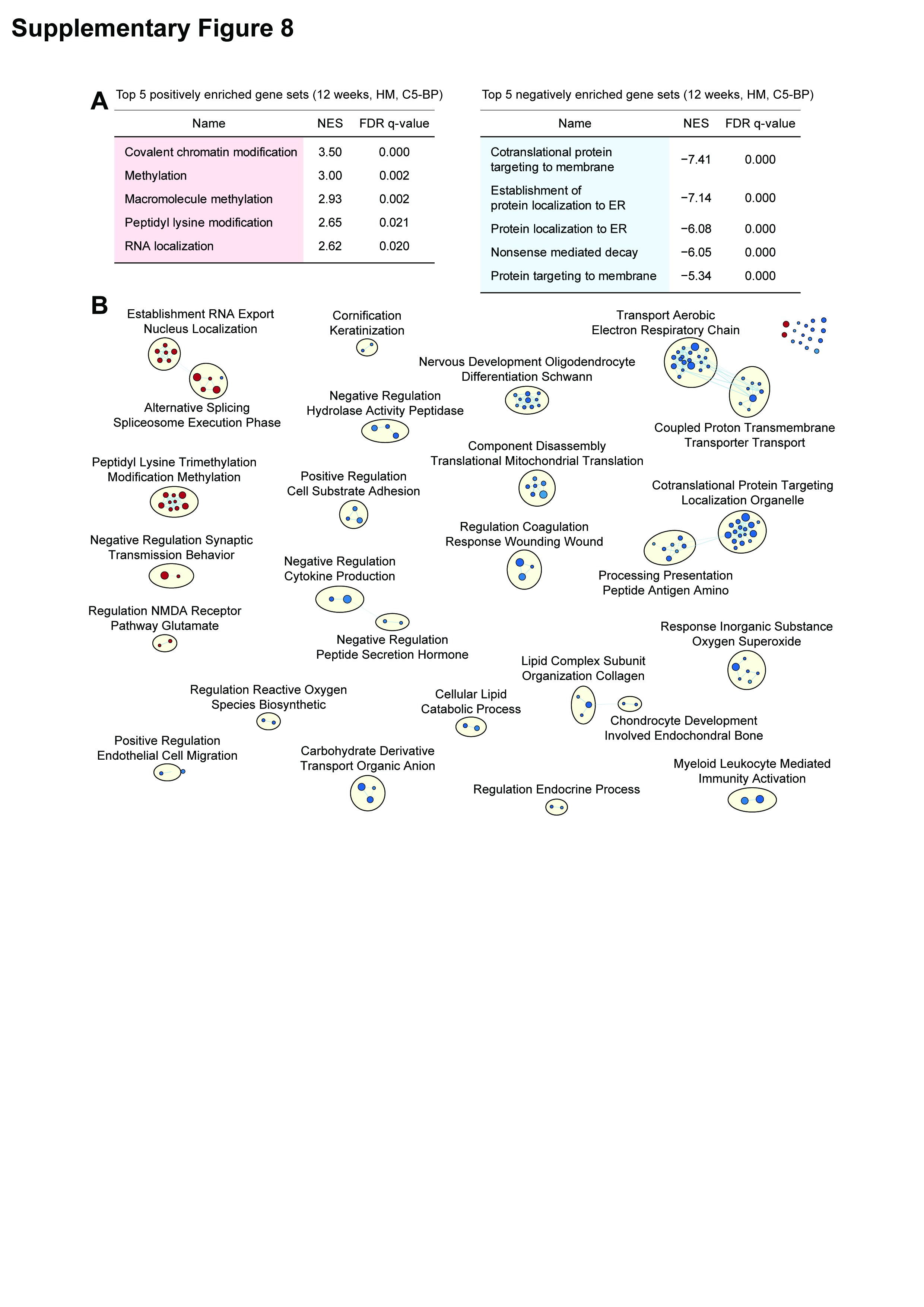

Supplement: Supplementary Figure 8 — GSEA of transcriptomes from W12-HM Shank2-mutant mice for biological functions in the C5-BP (biological process) domain. (A,B) GSEA results for W12-HM transcripts (A) and their integrated visualization generated using Cytoscape EnrichmentMap App (B) (n = 4 mice for WT, HT, and HM, FDR < 0.05). [file Image_8.TIF]

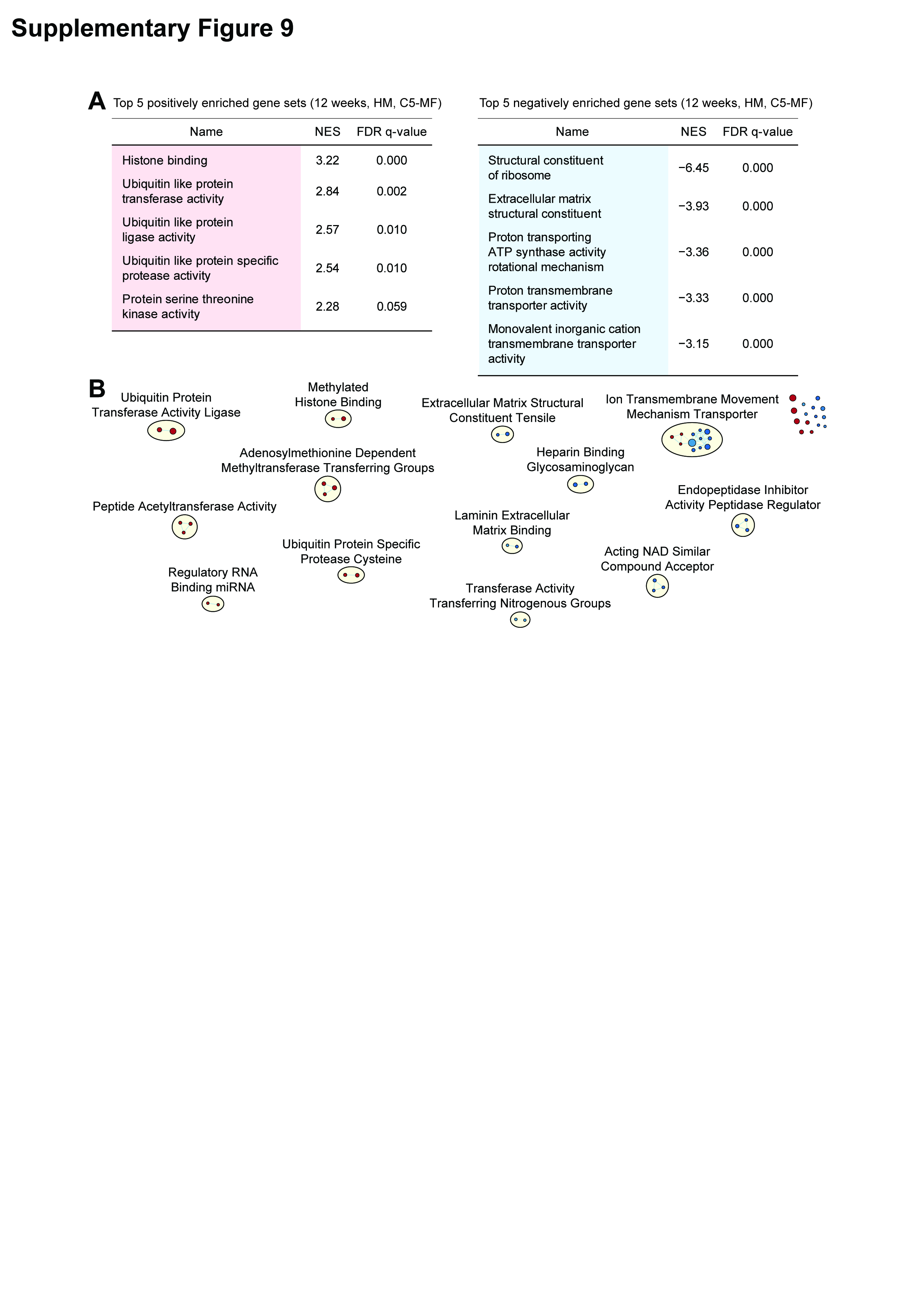

Supplement: Supplementary Figure 9 — GSEA of transcriptomes from W12-HM Shank2-mutant mice for biological functions in the C5-MF (molecular function) domain. (A,B) GSEA results for W12-HM transcripts (A) and their integrated visualization generated using Cytoscape EnrichmentMap App (B) (n = 4 mice for WT, HT, and HM, FDR < 0.05). [file Image_9.TIF]
